# Incidents of snake fungal disease caused by the fungal pathogen *Ophidiomyces ophidiicola* in Texas

**DOI:** 10.3389/ffunb.2023.1064939

**Published:** 2023-02-23

**Authors:** Alan J. Lizarraga, Lezley Hart, R. Michele Wright, Lance R. Williams, Joseph S. Glavy

**Affiliations:** ^1^ Biology Department, The University of Texas at Tyler, Tyler, TX, United States; ^2^ The Department of Pharmaceutical Sciences, Fisch College of Pharmacy, The University of Texas at Tyler, Tyler, TX, United States

**Keywords:** snake fungal disease, *Ophidiomyces ophidiicola*, Texas, fungal Infection, UV fluorescence

## Abstract

The pathogen *Ophidiomyces ophidiicola*, widely known as the primary cause of snake fungal disease (SFD) has been detected in Texas’s naïve snakes. Our team set out to characterize *O. ophidiicola’s* spread in eastern Texas. From December 2018 until November 2021, we sampled and screened with ultraviolet (UV) light, 176 snakes across eastern Texas and detected 27*. O. ophidiicola’s* positive snakes using qPCR and one snake in which SFD was confirmed *via* additional histological examination. Upon finding the ribbon snake with clear clinical display, we isolated and cultured what we believe to be the first culture from Texas. This cultured O*. ophidiicola* TX displays a ring halo formation when grown on a solid medium as well as cellular autofluorescence as expected. Imaging reveals individual cells within the septated hyphae branches contain a distinct nucleus separation from neighboring cells. Overall, we have found over 1/10 snakes that may be infected in East Texas, gives credence to the onset of SFD in Texas. These results add to the progress of the disease across the continental United States.

## Introduction

Across the globe fungal diseases are having large adverse effects on organisms. Fungal pathogens such as *Batrachochytrium dendrobatidis* (BD or Chytrid Fungus) in amphibians and *Pseudogymnoascus destructans* (pathogen in White Nose Syndrome) in bats have been shown to devastate large populations of organisms (Blehert et al., 2009). *Ophidiomyces ophidiicola* (*O. ophidiicola*) has been problematic with specific snake populations, including Eastern Massasaugas Rattlesnakes and Timber Rattlesnakes losing significant portions of their numbers (Allender et al., 2011; Fisher et al., 2012). *Ophidiomyces ophidiicola* has been confirmed as the primary pathogen in Snake Fungal Disease (SFD) (Rajeev et al., 2009). It has been identified throughout the Atlantic coast and into the Midwest of North America (Paré and Sigler, 2016; Davy et al., 2021; Vivirito et al., 2021). *O. ophidiicola* can spread to subdermal tissues causing the host discomfort and possible negative behavioral changes. Furthermore, individual snakes that do not display clinical signs of the disease can have their normal skin microbiota altered (Allender et al., 2020). These subdermal infections are characterized by lesions on the head, nose, body, and/or tail caused by dysecdysis and may cause face swelling, clouding of the eye, as well as fluid-filled vesicles (granulomas) (Lorch et al., 2015).

Other effects from SFD include increased basking, and decreased appetite. These are congruent with common symptoms among snakes fighting a variety of infections. Increased basking helps decrease the time between shedding events. These shedding events are a way that reptiles use their innate immune defense to get rid of skin infections (Stromsland and Zimmerman, 2017). While these processes may help snakes to recover, these modifications make snakes easier targets for predation and more likely to die of malnutrition (Lorch et al., 2015).

When we initiated our study, no known cases of SFD were verifiably linked with the primary pathogen (*O. ophidiicola*) found in Texas. However, there have been anecdotal sightings of symptomatic individuals in East Texas, and fungal infections found associated with Fusarium fungus in Southern Texas (Barber et al., 2016). The nearest researched case of SFD was in eastern Louisiana, making it a priority to monitor the pathogen’s spread westward (Glorioso et al., 2016). Harding et al. (2022) recently reported *O. ophidiicola* from water snakes in the Brazos river drainage of north central Texas.

With the addition of climate change increasing the temperature of the planet, these “seasons” will become longer. Increases in temperature will extend the viability of the fungi and geographic range (McKenzie et al., 2019). The purpose of this study is to survey snakes across east and into parts of central Texas to determine if there is a presence of *O. ophidiicola.* This data will be used to track the spread of the pathogen; therefore, determine its prevalence and at-risk populations. We hypothesized that there is a presence of snake fungal disease in Texas. If we detect *O. ophidiicola*, then our objective will be to isolate and culture the fungi to begin its characterization.

## Materials and methods

### Encountering snakes

From December 2018 to November 2021, various methods including shade traps, minnow traps, road cruising, walking and through contact with public (i.e., found snake on their property), were used to create encounters with snakes. Research was conducted in multiple counties across East and Central Texas. We collected samples in Smith, Freestone, Cherokee, Hunt, Navarro, Bell, Washington, Harris, Madison, and Henderson counties in Texas. We also sampled the Neches River Federal Wildlife Refuge (NRFWR) and the Old Sabine Bottom Wildlife Management Area (OSBWMA). Shade traps were set out at all locations; however, we reserved minnow traps to closer properties. Doing this we heavily reduced the chance of mortality caused by exposure or drowning. We also processed any snakes found on road dead (F.O.R.D.), or sheds we encountered.

Once a snake was encountered, we took down its GPS location, time, date, cover type, the position of the snake and noted any lesions. Additionally, we used UV flashlights in low light to examine for fluorescence similar to the Wood’s Lamp testing (Sharma and Sharma, 2016). However, instead of the 395nm that is usually used in a Wood’s Lamp test we used 365nm wavelength. Vivitrio et al. found in 2021 that the *O. ophiodiicola* fungal infections often gave off a UV fluorescence at 365nm and it was our aim to examine that as an indicator as well (Vivirito et al., 2021). Lesions were noted if they were comparable to snakes experimentally challenged in a study by Lorch et al. in 2015 (Lorch et al., 2015). These lesions are defined as thin scales with discoloration, ulcerations, and impacted scales (dysecdysis). Each individual snake was swabbed on the head and then on the body. We swabbed the head of the snake five or more times and down their ventral and lateral side. We did this using one swab for the head and one for the body (Veilleux et al., 2020). Any lesions that were found on the snake were photographed and individually swabbed. The swabs were stored in a 1.5- or 2-mL centrifuge tube at -20°C until DNA extraction. Any snakes that were handled without gloves on were removed from the data set (n=3, all *O. ophidiicola* negative).

Nitrile gloves were worn to avoid inadvertent spread of pathogens during handling of snakes. Lysol wipes and 10% bleach solution were used to disinfect equipment and boot bottoms between snakes and sites. Additionally, we cleaned and washed clothing that was worn on each sampling trip as another safeguard to prevent the spread of the pathogen. The snakes were not clipped or implanted with marking devices to protect them from unintentional pathogen introduction to their skin. The primary objective of this research is to determine if the disease has spread to Texas and any positive samples are important, regardless of recapture.

### Verification *via* quantitative PCR

DNA extraction of the swabs were initiated by soaking them in 1mL of lyticase (300U/mL) in microcentrifuge tubes for 24 hours to help break down the chitin walls of any fungi on the swabs (Bohuski et al., 2015). After the lyticase soak they were put through two freeze/thaw cycles and then centrifuged at 20,000 rpm for an hour to ensure all materials possible were removed from the swabs into the lyticase solution. The swabs were then removed from the remaining solution carefully and placed in another clean microcentrifuge tube and stored in a -80°C freezer for storage. We then put the solution through an extraction (Qiagen DNeasy Blood/Tissue Extraction Kit, Qiagen #69504) using the instructions provided by the kit. The purity and quantity of DNA extracts were determined by nanodrop spectrometry and aliquots of extracted samples were stored at -80°C for further analysis. We used a CFX Connect Real-Time PCR Machine (Bio-Rad, USA) to complete the quantitative polymerase chain reactions (qPCR) to amplify the fungal DNA. The *O. ophidiicola* specific primers used targeted the internal transcribed region (ITS) of ribosomal polymorphisms. These sequences are Oo-rt-ITS-F (forwardprimer) 5′-GAGTGTATGGGAATCTGTTTC-3′ and Oo-rt-ITS-R (reverse primer) 5′-GGTCAAACCGGAAAGAATG-3′ (Bohuski et al., 2015). We optimized our protocol using negative template and positive control specimens purchased from the University of Alberta Microfungus Herbarium (UAMH) Centre For Global Microfungal Biodiversity (#10769, UAMH). We used the qPCR settings from the methods found in research by Allender et al., 2015 using SYBR Green (Allender et al., 2015).

### Culturing of *O. ophidiicola*

*O. ophiodiicola* was inoculated on Sabouraud’s Dextrose Agar (SDA) plates and grown under gentamicin and chloramphenicol selection (50 μg/ml each) and grown at room temperature. Analysis of growth patterns were performed with inoculation in the center of plates. For liquid culturing, O*. ophidiicola* FL (#10769, UAMH) and *O. ophidiicola* TX were placed in yeast extract peptone dextrose (YPD) and shaker incubated (50 rpm) to test for growth at 37°C with or without gentamicin and chloramphenicol selection. Under these conditions, the fungi formed mainly hyphae in a few days.

### Ultraviolet measurements of cultured *O. ophidiicola*

Equal amounts of *O. ophidiicola* FL and *O. ophidiicola* TX culture was mixed and suspended in DNA loading buffer. Samples and a buffer alone control were loaded into separate wells of 1% agarose gel without addition of ethidium bromide. To measure individual fungus autofluorescence, wells were visualized with a standard UV transilluminator (365nm).

### Fluorescence and confocal microscopy

In our effort to visualize the autofluorescence of the fungi, *O. ophidiicola* were grown in poly-lysine coated 2-well chamber slides (Lab-Tek II, #154461) at 37°C in yeast extract peptone dextrose (YPD) media. Media was removed from each chamber and washed twice with filtered, sterile phosphate-buffered saline (PBS) ((pH7.4) contains 137 mM NaCl, 2.7 mM KCl, 8 mM Na_2_HPO_4_, and 2 mM KH_2_PO_4_). Fungi were fixed in 2% (*v/v*) formaldehyde/PBS for 10 min at room temperature, then washed with PBS and mounted onto slides using ProLong Gold Antifade reagent (Invitrogen P36934). Samples were analyzed at (ex 358nm, em 460nm) using either Echo Revolve Fluorescence microscope or our Zeiss LSM 880 confocal microscope with Airyscan and Zen processing (Zen Blue).

To visualize the nuclei of *O. ophidiicola* and due to overlapping emissions, nuclear orange was used. Nuclear orange LCS1 (AAT Bioquest, #17541) is a non-fluorescent DNA-selective and cell-permeant dye (ex 514nm, em 556nm). Nuclear orange has its fluorescence significantly enhanced upon binding to DNA, reducing background. *O. ophidiicola* was grown in poly-lysine coated 2-well chamber slides as above and then labeled with nuclear orange at 10 μM for 30 minutes. The media was removed and gently washed three times with PBS. After reducing moisture levels, slipcover mounting was performed with ProLong Gold Antifade reagent. Samples were analyzed using a Zeiss LSM 880 with Airyscan 5 and Zen processing (Zen Blue).

## Results

**Figure 1:**
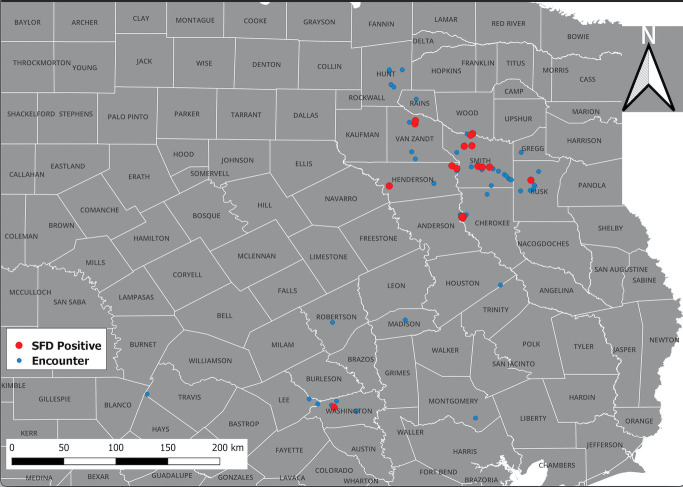
Distribution of *O. ophidiicola* in Texas. Texas State and locations positive for *O. ophiodiicola* are shown as red dots on the map (**Table 1**).

A total of 176 snakes were encountered during the project spanning 16 species, and a total 27 were positive for the fungal pathogen *O. ophidiicola* (**Figure 1**; **Table 1**). Using the presence/absence model from Hileman et al., 2018, we have categorized these snakes into three groups: SFD Positive (clinical and *O. ophidiicola* positive), Early Stage/Exposed (no clinical signs but *O. ophidiicola* positive), or No SFD/Failed to Detect (anything with or without clinical signs that lacked presence of *O. ophidiicola*) (**Table 2**) (Hileman et al., 2018). Three snakes were removed from the data set due to improper handling.

**Table 1:** Encountered species.

SPECIES	NUMBER ENCOUNTERED	O.O. POSITIVE
ELAPHE OBSOLETUS	35	2
AGKISTRODON PISCIVOROUS	20	4
NERODIA ERYTHROGASTER	20	4
SHED	17	0
THAMNOPHIS PROXIMUS	15	4
NERODIA RHOMBIFER	14	1
STORERIA DEKAYI	14	2
AGKISTRODON CONTORTRIX	13	2
MASTICOPHIS FLAGELLUM	6	2
HALDEA STRIATULA	5	2
NERODIA FASCIATA	5	1
FARANCIA ABACURA	2	0
LAMPROPELTIS CALLIGASTER	2	0
OPHEODRYS AESTIVUS	2	0
UNKNOWN*	2	0
LAMPROPELTIS HOLBROOKI	1	0
MICRURUS TENER	1	0
THAMNOPHIS SIRTALIS	1	0
VIRGINIA VALERIAE	1	0
TOTAL	176	27

Table showing numbers of encountered and qPCR positive snakes by species (O. ophidiicola = O.o.). *Unknowns are snakes that were deceased and unable to tell the species due to degradation.

**Table 2:** Severity of infection.

Positive for O.o.		Negative for O.o.		SFD	Early stage/Exposed	No SFD/Failed to detect
Clinical +	Clinical -	Clinical +	Clinical -	21	10	145
21	10	6	139			

Table showing infections delineated by metrics created by Hillman et al., 2018 (O. ophidiicola = O.o.). Using clinical and qPCR knowledge to sort snakes into three main categories of SFD, Early stage or Exposed, and No SFD or Failed to detect.

**Figure 2:**
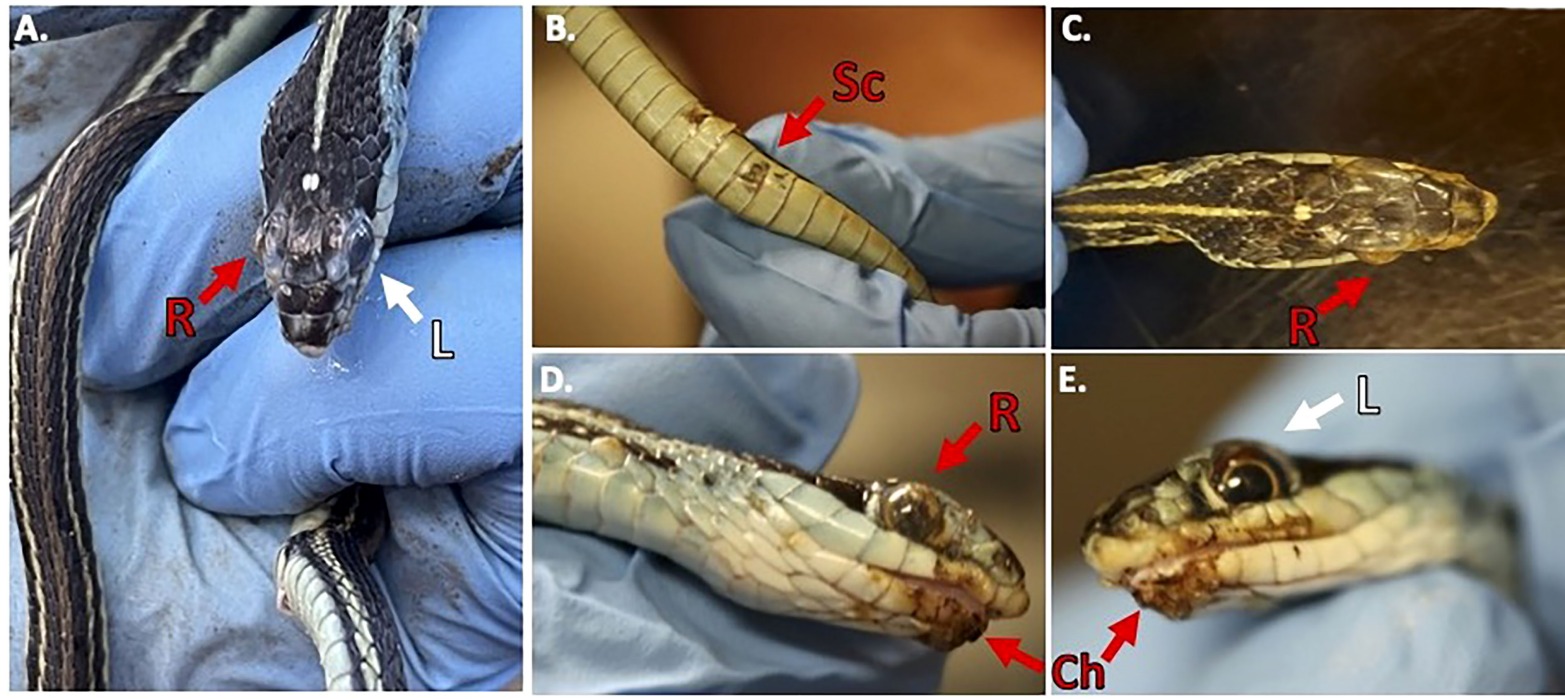
Clinical Signs of Snake Fungal Disease in the Field. Photos of collected Western Ribbonsnake (Thamnophis proximus). Panel **(A)**, comparison between the gross lesions on the right eye (“R” in red), and unaffected eye (“L” in white). Panel **(B)**, lesions on ventral scales of the specimen (“Sc” in red). Panel **(C)**, over the top view of lesions on head. Panel **(D)**, side view of right eye lesions and panel **(E)**, View of gross lesions under the chin (“Ch” in red) of the specimen (Snakes handled by first author, Alan Lizarraga, granted permission for photo).

During our encounters, we found a Western Ribbonsnake (*Thamnophis proximus*) that was in a condition to legally collect and have processed for histology. The snake was found at the Old Sabine Wildlife Management Area (OSBWMA), it had mild to moderate crust on its swollen right eye and chin, multiple areas of crusted/missing scales along the ventral side of its body, and dysecdysis on its dorsal side (**Figure 2**). The snake was moribund and was housed according to Institutional Animal Care and Use Committee (IACUC) requirements. However, it died approximately two days after collection. Prior to death, we were able to culture fungi (*O. ophidiicola* TX) from its sores and confirmed *via* qPCR that it was *O. ophidiicola* (see methods: *O. ophidiicola* specific primers) (Bohuski et al., 2015).

**Figure 3:**
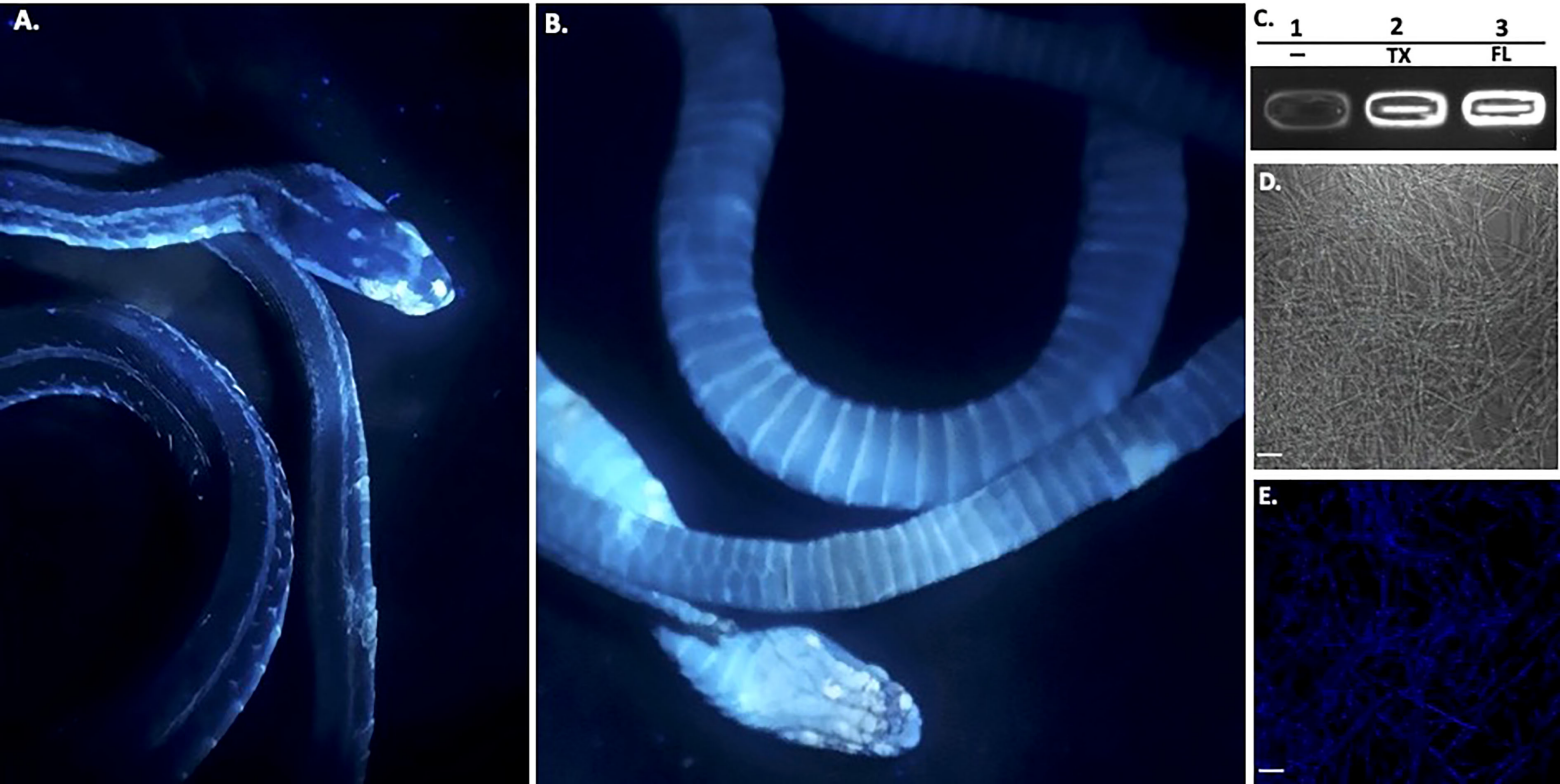
Ultraviolet Fluorescence. The infected deceased Western Ribbonsnake from **Figure 2** under UV light (365nm) lamp, images shown are dorsal side **(A)** and ventral side **(B)**. Areas of intensity signal are evidence of the pathogen, *O. ophiodiicola*. **(C)**, Transilluminator readings of UV fluorescence between strains *O. ophidiicola FL* (UAMH #10769) and *O. ophidiicola* TX. Lane 1, Buffer alone, lane 2, *O. ophidiicola* TX, Lane 3, O*. ophidiicola* FL. **(D)**, Confocal microscope imagery of *O. ophidiicola* TX in light phase and Texas found strain. **(E)**, UV fluorescence after excitement at 358 nm and its emission at 460 nm (blue). Bars: **(D, E)** = 30 μm.

The snake was then photographed under UV light (**Figure 3**). The samples were stored at -20°C until the specimen was delivered to the United States Geological Survey (USGS) for histological analysis. This snake was sent to the U.S. Geological Survey - National Wildlife Health Center (Madison, WI) for processing where *O. ophidiicola* was isolated in culture from skin lesions and SFD was confirmed histopathologically (**Supplemental Material A**). Additionally, the analysis found that tapeworms had also infected the snake. We do not know if this happened before or after the infection of the snake by the pathogen. Furthermore, the U.S. Geological Survey - National Wildlife Health Center (Madison, WI) forwarded the IT’S sequencing data generated from this *O. ophidiicola* (**Supplemental Material B**, 30523_1_I1_ITS, 610bp). The ribosomal internal transcribed spacer (ITS) region is the most commonly chosen genetic marker for the molecular identification of fungi (Nilsson et al., 2015). We found *O. ophidiicola* TX is 100% identical to *O. ophidiicola* FL (#10769 UAMH, KF477235 ITS, 586 bp) in the ITS region.

Long-wave ultraviolet (UV, 365nm) light can detect fungal skin infections and is utilized as a field detection screen tool (Turner et al., 2014; Breyer et al., 2021; Vivirito et al., 2021). Upon UV exposure, *O. ophidiicola* positive snakes emit a UV fluorescence, as shown with the infected Western ribbon snake from **Figure 2** (**Figures 3A, B**). The intensity of the signal correlates with the amount of fungus at each site (Turner et al., 2014; Vivirito et al., 2021). To further examine this emission, we loaded *O. ophidiicola* cultures into the wells of unstained agarose gel. Both *O. ophidiicola* TX and *O. ophidiicola* FL (**Figure 3C**, lanes 2&3) transmit a detectable signal compared to buffer alone (lane 1) when placed on a standard UV transilluminator (Sung-Jan Lin et al., 2009; Breyer et al., 2021). Furthermore, this ultraviolet fluorescence is detected at the cellular level (**Figure 3D** (light field) & E (UV fluorescence), as shown with confocal imaging of natural transmission as seen in other fungi including oceanic fungi (Sung-Jan Lin et al., 2009; Breyer et al., 2021). UV fluorescence may be the result of high content of chitin and/or ergosterol in fungal species (Turner et al., 2014; Breyer et al., 2021).

**Figure 4:**
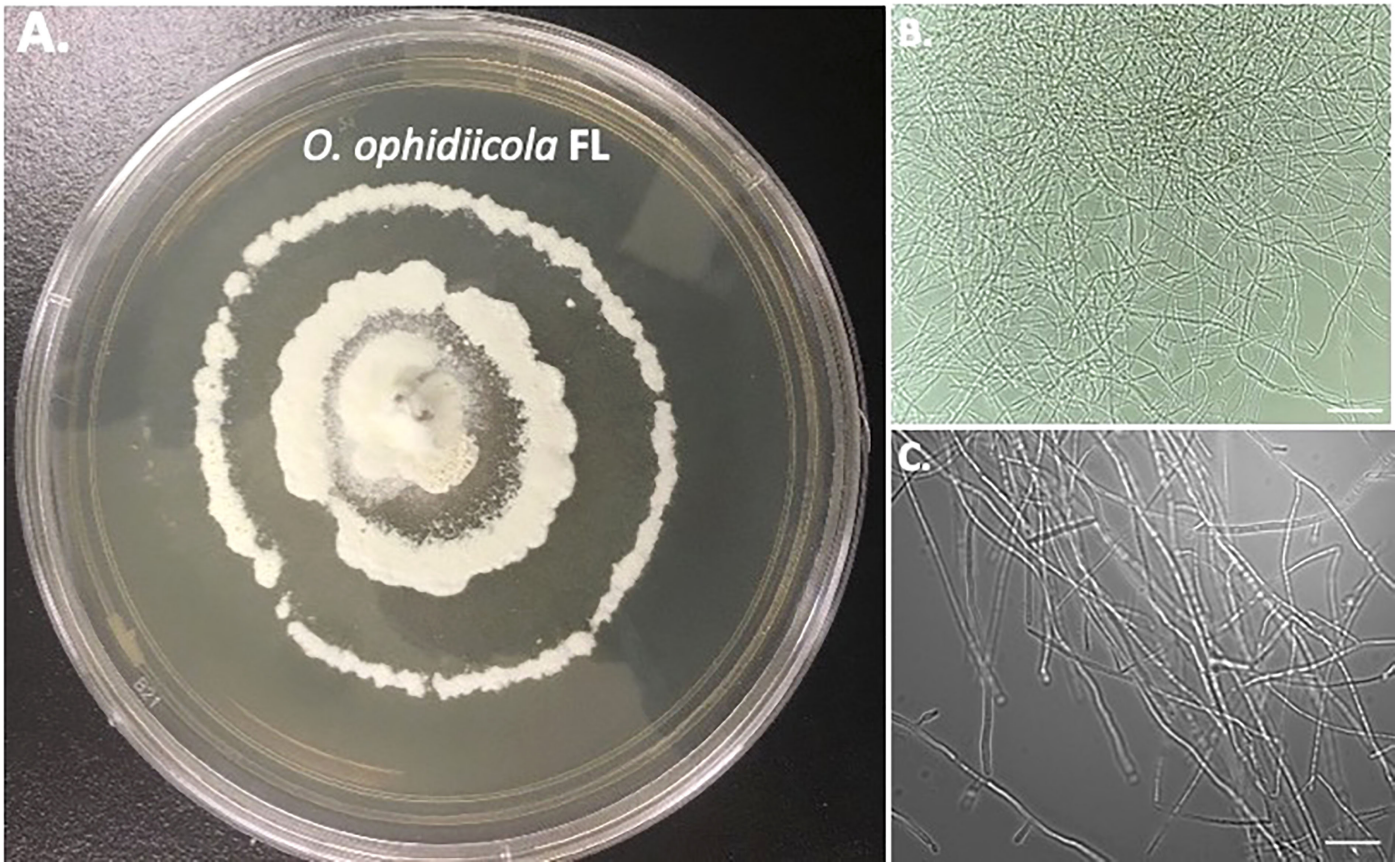
Colony and Microscopic Features of *O. ophidiicola* FL. Comparison of colony formation SDA plates: *O. ophidiicola* FL **(A)** Light microscope images of hyphae phase: *O. ophidiicola* FL **(B, C)**. Bars: B = 150 μm; C = 75 μm.

**Figure 5:**
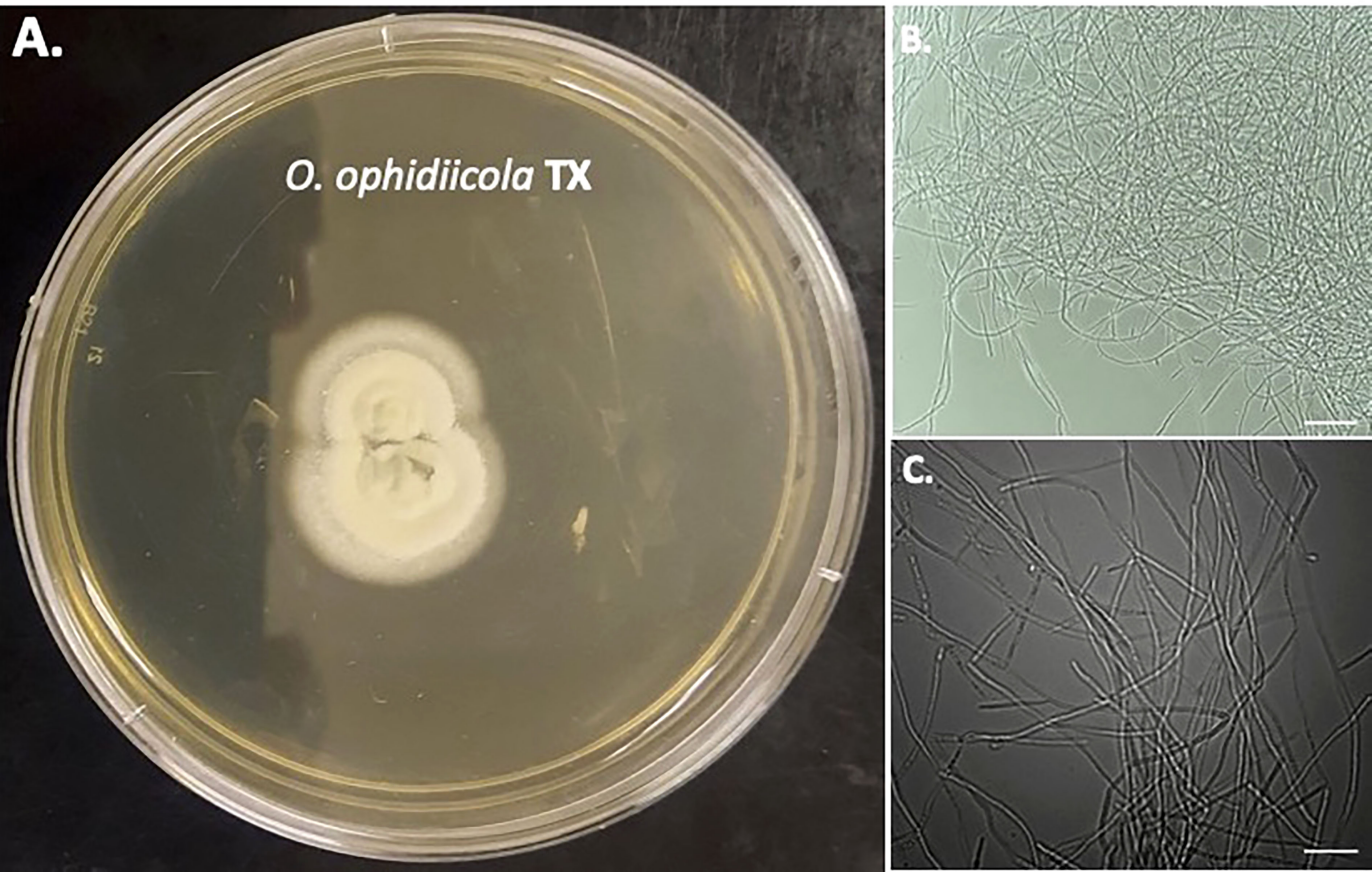
Colony and Microscopic Features of *O. ophidiicola* TX. Comparison of colony formation SDA plates: *O. ophidiicola* TX **(A)** Light microscope images of hyphae phase: *O. ophidiicola* TX **(B, C)**. Bars: B = 150 μm; C = 75 μm.

Furthermore, a swab from the infected right eye (**Figure 4D**) was plated on Sabouraud’s Dextrose Agar (SDA) plates and grown at room temperature (See methods). **Figures 4**, **5** shows the growth of *O. ophidiicola* FL (**Figure 4A**) and *O. ophidiicola* TX (**Figure 5A**). Plates were inoculated in the center and allowed to grow for 25 days (about 3 and a half weeks)*. O. ophidiicola* FL forms a unique bullseyes growth pattern, while *O. ophidiicola* TX stayed in the center with ring halos forming its growth strata (**Figures 4A**, **5A**). Examination under a light microscope reveals both are forming a uniform hyphae (mycelium) (**Figures 4B, C**, **5B, C**).

**Figure 6:**
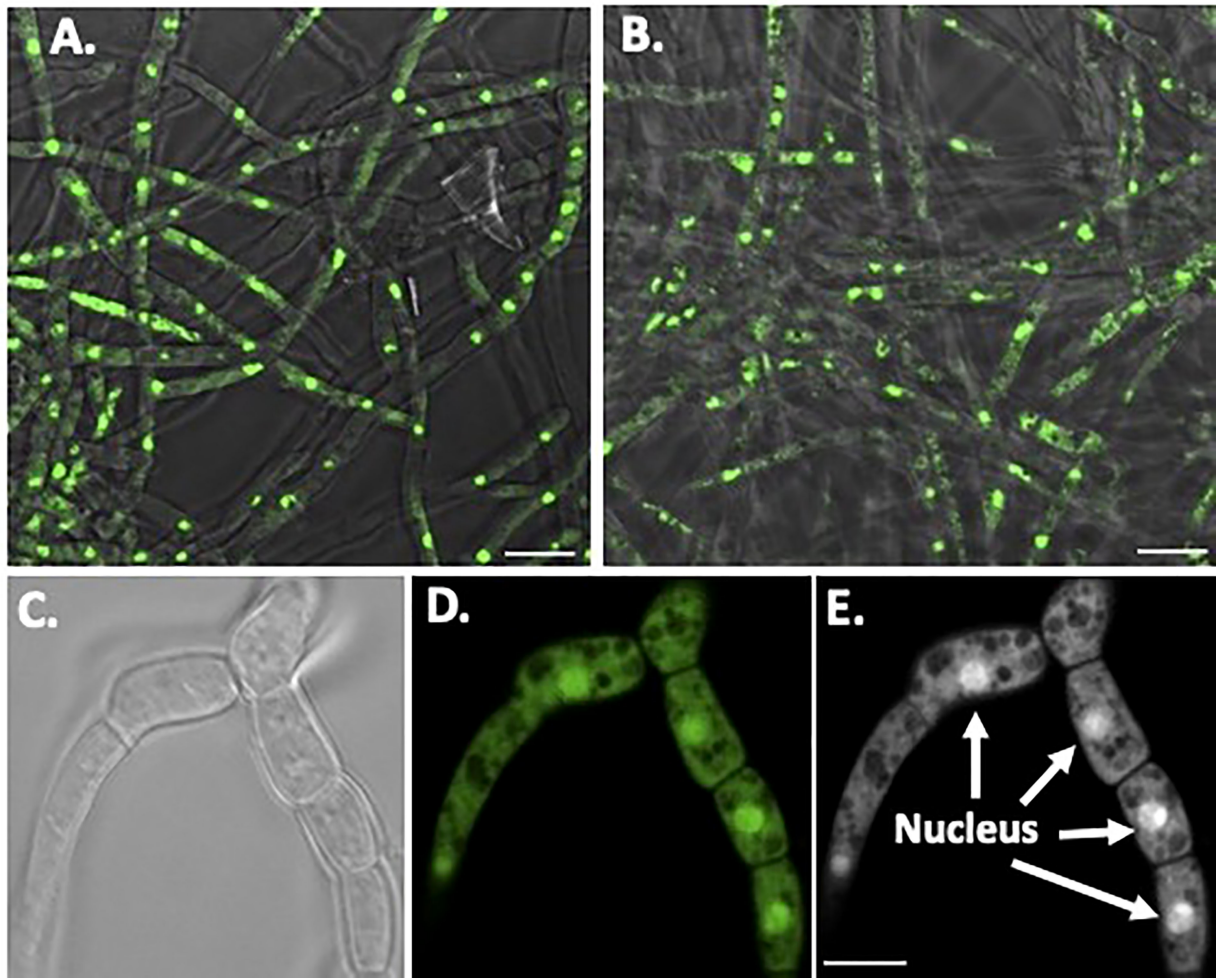
Nuclear Staining of O. ophidiicola Hyphae. Comparison of merged bright transparent field and fluorescence nuclear staining (Green) between O. ophidiicola FL panel **(A)** and O. ophidiicola TX panel **(B)**. Lower set of panels show the hyphae branch point of O. ophidiicola TX first in the light field panel **(C)** then nuclear orange staining panel **(D)**. Black and white of nuclear staining and defined septate hyphae panel **(E)**. Bars: A and B = 10 µm; C, D, and E = 5 µm.

Nuclear orange staining reveals the nucleus within each cell without UV fluorescence interference (**Figures 6A, B**). Overall, *O. ophidiicola* FL and *O. ophidiicola* TX, have the same nuclei pattern throughout their hyphae. Individual cells within the septated hyphae branches contain a distinct nucleus separation from neighboring cells as seen in **Figure 6C**, panel C (light field) & **Figure 6**, panel D (nuclear orange stained nuclei, dense green). Distinct dense nuclear regions of individual cells are noted in negative panel (**Figure 6E**). The nucleus *of O. ophidiicola* contains differentiated areas corresponding to chromatin packing and separated condensed packed section consistent with its nucleolus. These results consistently show a single nucleus for each hyphae cell.

## Discussion

SFD has been found in many states in the USA, ranging from the eastern seaboard to Idaho, as well as newly found cases in California (Allender et al., 2020; Haynes et al., 2021). A study from the Brazos River Basin in north central Texas (Harding et al., 2022) identified *O. ophidiicola* from water snakes in the Brazos river drainage. Almost half of the aquatic snakes tested in the Brazos River Basin were positive for SFD. Our study included both positives for aquatic and terrestrial snakes. The prevalence of *O. ophidiicola* in our study for the total population was 15% of the 176 sampled snakes. The results of our study showed that 15% of the 39 sampled aquatic snakes tested positive for the fungus, while 17.8% of the 118 terrestrial snakes tested positive. These studies are a preliminary comparison of the ecosystem around the Brazos River Basin to the multiple types of ecosystems in East-Central Texas where our research was conducted. In a broader targeted surveillance in the western US and Puerto Rico, 54% of the 35 aquatic snakes tested positive for *O. ophidiicola*, while 17.6% of the 528 terrestrial snakes tested positive (Allender et al., 2020). Further research needs to be conducted on the prevalence of SFD in terrestrial vs. aquatic species in different ecosystems.

Using UV to aid detection will be a helpful tool; we can make these correlations easier and sooner with more data. The ultraviolet light aspect of this fungus adds to the ability to monitor pathogens and is necessary to establish control/mitigation strategies. UV fluorescence is present in yeast and other fungi at the cell level responsible for skin infections and in forms of marine fungi (Maslanka et al., 2018; Breyer et al., 2021). The origin of this fungal autofluorescence may result from a high content of chitin or ergosterol in fungal species (Turner et al., 2014).

The moribund ribbon snake has supported our hypothesis that SFD caused by *O. ophidiicola* has made it to Texas and has been confirmed using three different avenues, plus ITS sequencing data of the moribund ribbon snake (**Supplemental Material B**, 30523_1_I1_ITS, 610bp). *O. ophidiicola* TX is a match to *O. ophidiicola* FL (UAMH 10769, KF477235 ITS, 586 bp). Our successful culturing of *O. ophidiicola* TX will allow us to characterize the cell structures and ultra-structure further and then compare them to other cultured *O. ophidiicola* from various locations. *O. ophidiicola* FL*’s* unique bullseye growth pattern and *O. ophidiicola* TX’s ringed halo pattern initially distinguish the two (**Figures 4A**, **5A**). Further examination under the light microscope unveils a uniform mycelium pattern (**Figures 4B, C**, **5B, C**). Through nuclear orange staining, we show that each cell has a distinct nucleus and compartments (**Figure 6**). Their nuclear morphology is a simple circular shape centered midway in each cell, of which we estimate the nuclear diameter between 1.3 and 1.5 nm (**Figures 6C–E**). This data begins to characterize the septated hyphae and branch point arrangement of hyphae (**Figures 6C–E**).

After finding the pathogen in the same area for two consecutive years, we now have a spatiotemporal update on the natural reach of the disease. In future experiments, we will use our presence/absence data to investigate potential avenues of dispersal, primarily through the scope of climate change as the area gets more fungal-friendly (warmer and wetter). We have yet to determine the seasonality of its onset, but others have shown the condition to be more prevalent in early spring and late summer (McKenzie et al., 2019). Continuing surveillance on snake populations in Texas may give valuable data for documenting the spread.

This study focused solely on wild snake populations, but one of the primary means of invasive species introduction is the pet trade (Thompson et al., 2018); poor conditions and cramped enclosures are an incubator for pathogens, especially when the animals are a newer monoculture. By monitoring positive cases both in captivity and in the wild we can gain a better understanding of how anthropomorphic and natural based infections spread (Charron et al., 2013). Doing so will allow researchers time to produce solutions to combat this deadly fungus and other pathogens like it.

Further studies into soil, elevation, climate, and species preferences will also help us learn more about the fungal spread. The UV absorbance of this fungus adds to the ability to monitor pathogens and is necessary to establish control/mitigation strategies. These steps are all paramount when tracking the spread of SFD, and using these fields to bench methods, the more information we can gain in these areas, the more effective we will become at fighting the disease.

## Data availability statement

The original contributions presented in the study are included in the article/**Supplementary Material**. Further inquiries can be directed to the corresponding authors.

## Ethics statement

The animal study was reviewed and approved by Institutional Animal Care and Use Committee (IACUC), University of Texas at Tyler.

## Author contributions

The authors designed and performed the experiments, The corresponding authors wrote the manuscript and the rest of the authors finalize the manuscript. All authors contributed to the article and approved the submitted version.

## Supplementary material

Supplemental Material A United States Geological Survey (USGS) histological analysis stating SFD was confirmed histopathologically.

Supplemental Material B United States Geological Survey (USGS) ITS sequencing data confirming identity (30523_1_I1_ITS) and compared to *O. ophidiicola* FL (UAMH 10769, KF477235 ITS, 586 bp). Highlighted sequence is shared by both and 100% identical in ITS region.
