# Supplementary material for: Incidents of snake fungal disease caused by the fungal pathogen Ophidiomyces ophidiicola in Texas
Source: Front Fungal Biol. 2023 Feb 23;4:1064939. doi: 10.3389/ffunb.2023.1064939 (PMC10512329; doi:10.3389/ffunb.2023.1064939)
Supplement: Supplemental Material B — United States Geological Survey (USGS) ITS sequencing data confirming identity (30523_1_I1_ITS) and compared to O. ophidiicola FL (UAMH 10769, KF477235 ITS, 586 bp). Highlighted sequence is shared by both and 100% identical in ITS region. [file DataSheet_2.pdf]

U.S. Geological Survey - National Wildlife Health Center (Madison, WI)
ITS_18S ribosomal RNA gene
Identical Regions

Ophidiomyces ophiodiicola, TX
>30523_1_I1_ITS.
GTCGTAACAAGGTTTCCGTAGGTGAACCTGCGGAAGGATCATTACCGTGTTGGTTGA
GCGCCCCTCCCCCCCGGGTCCCCCGGGGGCCGGGCGCCCCTACCTCCACCCGTGTCT
ACCCCATTCCGTTGCCTCGGCGGGCCCGCCAGCCACCAGCCTGGCGGTCTCCGGGCG
TCCCCTCCTGGGGGCGGCCCCGGGGGCCCGCGCCCGCCGGTGGAACCGTGAACGAA
CTCTGTGAGAAGCCAGTCTGTCTGAGCACGAATGTGAAATCATGTAAAACTTTCAAC
AACGGATCTCTTGGTTCCGGCATCGATGAAGAACGCAGCGAAATGCGATAAGTAAT
GTGAATTGCAGAATTCCGTGAATCATCGAATCTTTGAACGCACATTGCGCCCCCTGG
TATTCCGGGGGGCATGCCTGTCCGAGCGTCATTGCAACCCCCTCAAGCCCGGCTTGT
GTGTTGGGGGTGCCCACCCCGAAGTCCTCGGGCGCGGGCCCCCCCCAAATGCAGTG
GCGGCACCGAGTTCCTGGTGTCTGAGTGTATGGGAATCTGTTTCTGTCTCGCTCGAA
GACCCGATCGGCGCCCGTCGTCAACCCCCCATTCTTTCCGGTT

UAMH 10769 *Ophidiomyces ophiodiicola*, FL
>KF477235 ITS

CATTACCGTGTTGGTTGAGCGCCCCTCCCCCCCGGGTCCCCCGGGGGCCGGGCGCCC
CTACCTCCACCCGTGTCTACCCCATTCCGTTGCCTCGGCGGGCCCGCCAGCCACCAG
CCTGGCGGTCTCCGGGCGTCCCCTCCTGGGGGCGGCCCCGGGGGCCCGCGCCCGCC
GGTGGAACCGTGAACGAACTCTGTGAGAAGCCAGTCTGTCTGAGCACGAATGTGAA
ATCATGTAAAACTTTCAACAACGGATCTCTTGGTTCCGGCATCGATGAAGAACGCAG
CGAAATGCGATAAGTAATGTGAATTGCAGAATTCCGTGAATCATCGAATCTTTGAAC
GCACATTGCGCCCCCTGGTATTCCGGGGGGCATGCCTGTCCGAGCGTCATTGCAACC
CCCTCAAGCCCGGCTTGTGTGTTGGGGGTGCCCACCCCGAAGTCCTCGGGCGCGGGC
CCCCCCCAAATGCAGTGGCGGCACCGAGTTCCTGGTGTCTGAGTGTATGGGAATCTG
TTTCTGTCTCGCTCGAAGACCCGATCGGCGCCCGTCGTCAACCCCCCATTCTTTCCGG
TTTGACCTCGGATCAGG
